# Functional sinonasal outcomes after rescue flap versus double nasoseptal flap in endoscopic trans-sphenoid pituitary surgery: a randomized clinical trial

**DOI:** 10.1007/s00405-025-09342-8

**Published:** 2025-04-12

**Authors:** Ahmad Muhammad Al-Arman, Waleed Moneir, Hazem Emam Amer, Mahmoud Saad, Hisham Atef Ebada

**Affiliations:** 1 Department of Otorhinolaryngology, Mansoura University, Mansoura, 35511 Egypt; 2 Department of Neurosurgery, Mansoura University, Mansoura, Egypt

**Keywords:** Pituitary adenoma, Trans-sphenoid, Rescue flap, Nasoseptal flap, Sinonasal, Outcomes

## Abstract

**Objectives:**

The aim of the current study was to evaluate the functional sinonasal outcomes after rescue flap versus double nasoseptal flap in endoscopic endonasal pituitary surgery.

**Methods:**

This randomized clinical trial was conducted over 1.5 years over 60 patients who underwent endoscopic trans-sphenoid surgery for macroadenomas (more than 2 cm.). the patients were randomly allocated into 2 groups: the rescue flap group, (*n* = 30) and the double nasoseptal flap group (*n* = 30). Functional sinonasal outcomes were evaluated in both groups in terms of sinonasal outcome test (SNOT-22), crusting, adhesions, and olfaction.

**Results:**

the sinonasal outcome test (SNOT-22), as well as the olfaction scores were significantly better in the double flap group compared to the rescue flap group. Crusting and adhesions occurred more frequently in the rescue flap group. The nasal stage operative time was significantly longer in the double flap group than the rescue flap group.

**Conclusion:**

Endoscopic pituitary surgery can adversely affect the sinonasal functions. Double nasoseptal flap technique allows posterior septectomy with bilateral septal mucosa preservation. Although it requires longer operative time than the rescue flap technique, better functional sinonasal outcomes and olfaction scores are achieved.

## Introduction

The endoscopic approach to the pituitary gland is regarded as minimally invasive when compared to the microscopic technique, as it does not involve a sublabial approach, transseptal incision, or speculum insertion. Consequently, this method results in lower overall complication rates, shorter operating room time, reduced hospital stays, and less patient discomfort [1, 2]. However, one of the major side effects of trans-sphenoid pituitary surgery is potentially a deterioration of subjective sinonasal functions [3].

In endoscopic endonasal pituitary surgery, posterior septectomy is commonly performed for accessing sphenoid sinuses, ensuring four-handed surgery, and increasing visualization. Additionally, extensive sphenoidotomy is usually performed leading to more mucosal loss. Due to excessive mucosal loss, the probability of developing septal perforation, olfactory loss, crusting, and sinusitis may increase [4].

Various techniques and flaps are adopted during endoscopic endonasal pituitary surgery. The primary objectives of these techniques are easy and adequate reconstruction with minimal damage to minimize the adverse effects on the sinonasal functions [4–6].

The nasoseptal flap has been widely used since its introduction in 2006 [7] and is considered the workhorse for reconstruction in endoscopic skull base surgery including endoscopic pituitary surgery [8]. Double nasoseptal flap technique was described by Gode et al. [9]. This technique depends on a fully harvested bigger nasoseptal flap on one side and smaller one on the other side. By placing the anterior and superior margins of incisions to different regions on the mucoperichondrium of the right and left sides, the two flaps could be re-sutured to their original places without any perforations at the end of the surgery [9].

The nasoseptal rescue flap was introduced because leakage of cerebrospinal fluid is not anticipated in the majority of pituitary tumors, and routine elevation of full nasoseptal flap may be unnecessary [10]. Rescue flap involves making only a horizontal incision which can be completed to a full nasoseptal flap when required. When not required, the flap is repositioned to the original site to decrease the donor site morbidity [11].

The aim of the current randomized clinical trial was to evaluate the functional sinonasal outcomes after rescue flap versus double nasoseptal flap in endoscopic endonasal pituitary surgery.

## Patients and methods

### Patients

This randomized clinical trial was performed in the Otorhinolaryngology department, and the Neurosurgery Department, Faculty of Medicine, Mansoura University, Egypt, over 1.5 years (January 2023– June 2024), on all consecutive patients who underwent endoscopic endonasal pituitary surgery for pituitary macroadenomas (more than 2 cm.). The study was approved by the Mansoura Faculty of Medicine Institutional Research Board (IRB: MD.22.11.721.R1). This randomized clinical trial was registered at ClinicalTrials.gov (NCT06526481).

The patients of the study were randomly allocated into groups using the block randomization method: group A (rescue flap group; *n* = 30) and Group B (double flap group; *n* = 30).

### Surgical techniques

All surgeries were done by the same surgical team (the authors of this work). After sterilization and draping, infiltration of nasal septal mucosa was done with 1% lidocaine with epinephrine in a 1/100,000 dilution. Inferior and middle turbinates were lateralized and out fractured to facilitate visualization of sphenoid ostium.

**Fig. 1:**
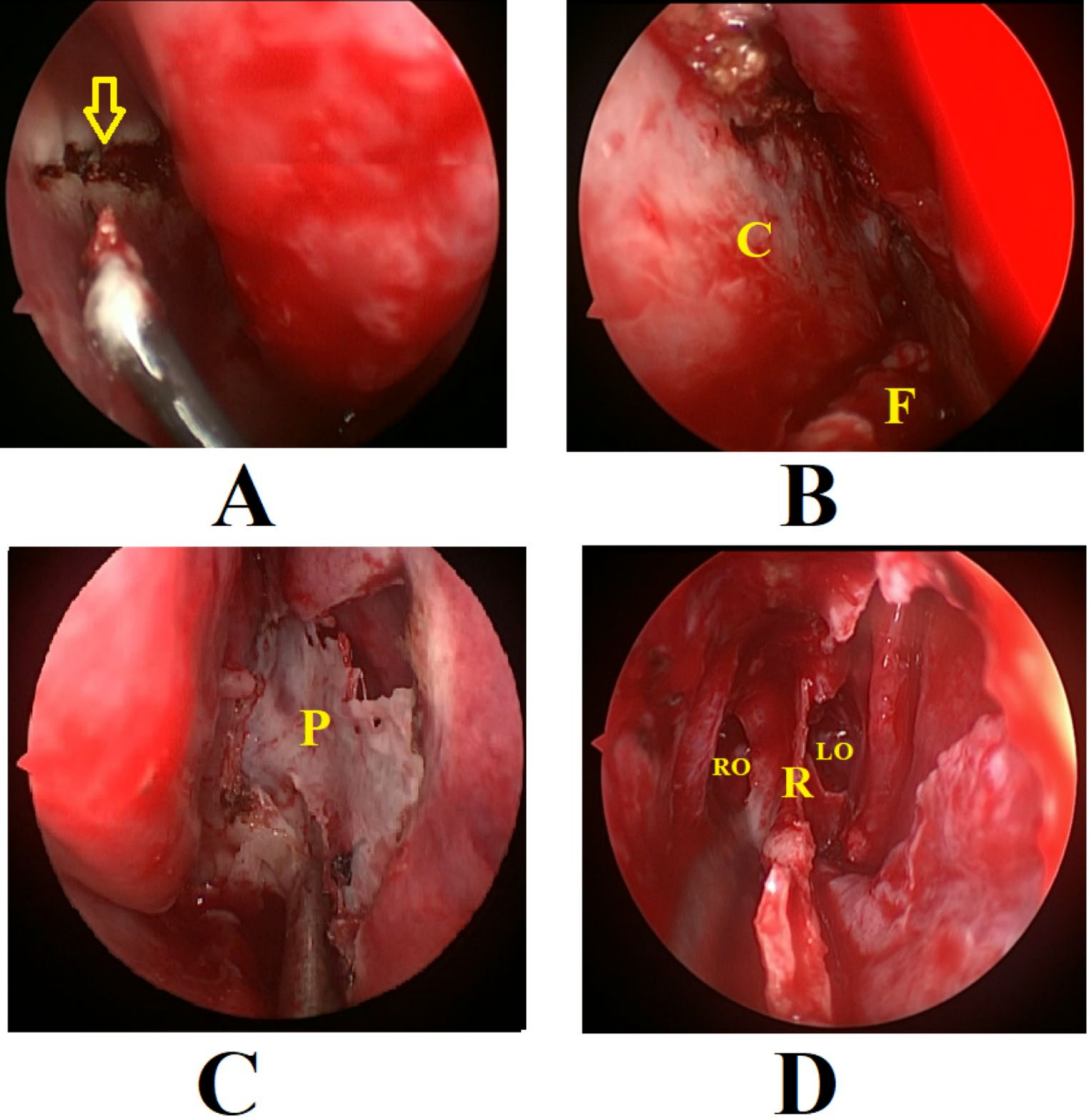
The rescue flap technique. **A**: The horizontal incision on the nasal septum (the arrow). **B**: Elevation of the flap (**F**) downwards from the septal cartilage. **C**: Posterior septectomy is performed by removing the perpendicular plate of the ethmoid (**P**) and vomer without preservation of the contralateral mucosa. **D**: Exposure of the sphenoid rostrum (**R**) and ostia; RO: right ostium, LO: left ostium

In the rescue flap group, a horizontal incision was made across the anterior face of the sphenoid, at the level of the sphenoid ostium. This incision was then extended medially over the sphenoid rostrum and carried anteriorly into the nasal septum, encompassing approximately one-third to one-half of the septum along the sagittal plane (Fig. 1: A).

The mucosa of anterior wall of sphenoid and septum was then dissected and retracted inferiorly preserving the pedicle of nasoseptal rescue flap (Fig. 1: B). Wide sphenoidotomy was one on the same side above the rescue flap pedicle, then a contralateral wide sphenoidotomy was also performed.

To obtain wide exposure and bi-nostril access, a posterior septectomy was performed, including the septal mucosa contralateral to the flap (Fig. 1: C). Exposure was expanded by removing the inter sphenoid septations. Sphenoid sinus mucosa was removed. The Sellar floor was removed, and the dura was incised. The pituitary adenoma was dissected and removed.

After tumor resection, in patients without CSF leak (*n* = 24), reconstruction was achieved by using surgicel, bone chips and free mucosal grafts. The nasoseptal rescue flap was returned and stitched in its original position. However, when there was CSF leak (*n* = 6), the rescue flap was extended to a full nasoseptal flap. The incision was extended anteriorly to the anterior end of the nasal septum, then from this point inferiorly to the nasal floor. Another horizontal incision was done from the choana along the nasal floor to meet the last point of the anterior incisions. The flap was elevated and rotated posteriorly to cover and reconstruct the sellar floor. The sphenoid sinus was packed with surgicel and gelfoam.

**Fig. 2:**
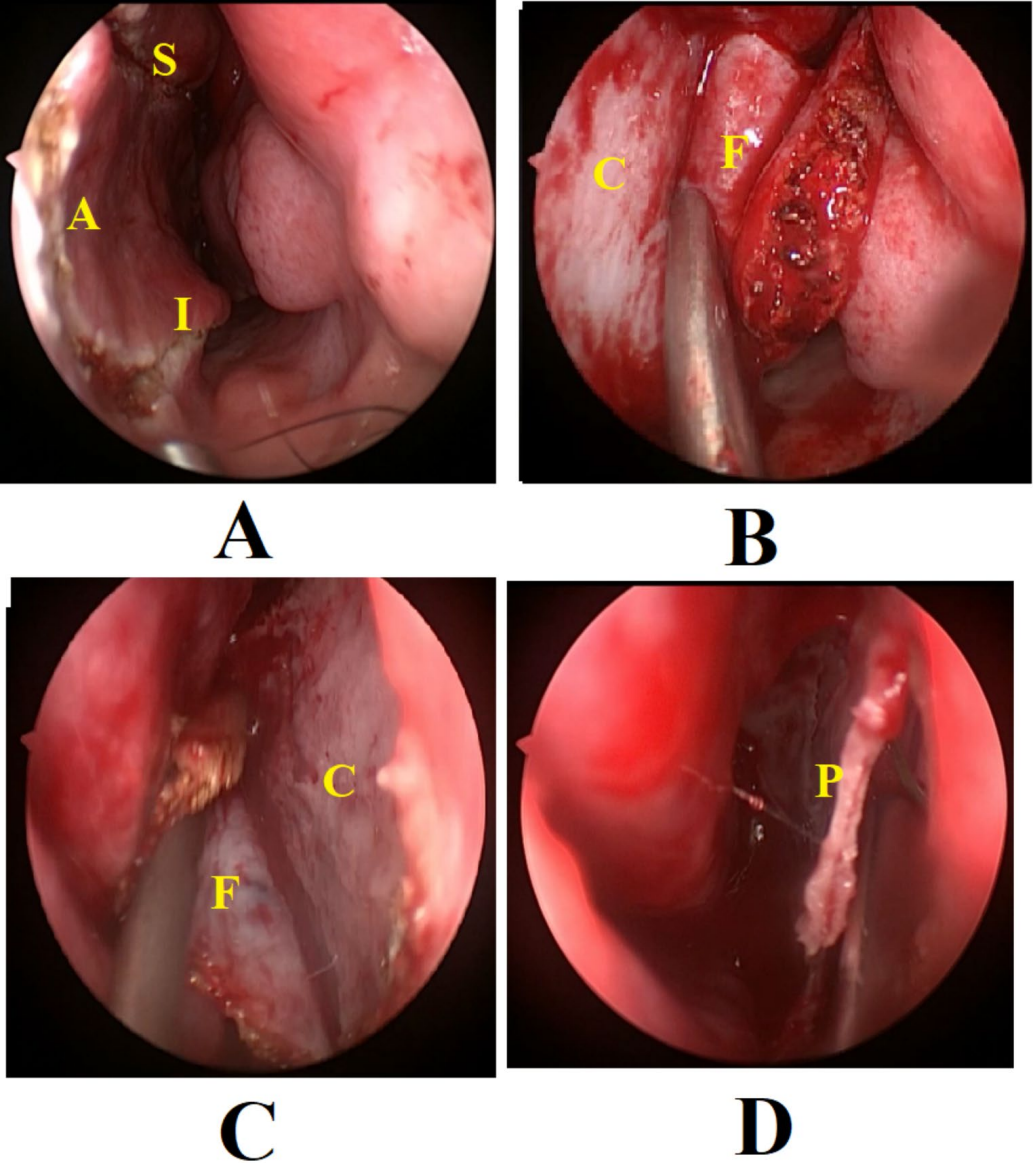
The double nasoseptal flap group. **A**: Incisions of the flap; the superior incision (**S**) and the inferior incision (**I**) are connected anteriorly with an anterior vertical limb (**A**). **B**: Elevation of the flap (**F**) from the septal cartilage (**C**). **C**: Elevation of a contralateral smaller flap. **D**: Posterior septectomy is performed after elevation of the two flaps; **P**: perpendicular plate of the ethmoid bone

On the other hand, in the double nasoseptal flap group, a full nasoseptal flap was performed on one side (usually the left side was preferred). The superior incision was placed at least 1 cm. below the skull base to preserve the olfactory mucosa. The anterior limit was 0.5 cm to the mucocutaneous junction of the nostril (Fig. 2: A). The flap was harvested (Fig. 2: B), rotated posteriorly and stored in the nasopharynx until the end of the procedure. On the other side of the nose, a shorter and narrower nasoseptal flap was performed (Fig. 2: C). The superior incision is about 1–2 cm. lower than the contralateral one, and similarly, the anterior incision is 1–2 cm. posterior to the contralateral one in order to achieve non-opposing incisions. This flap was elevated and retracted laterally to allow posterior septectomy without excision of the mucosa (Fig. 2: D).

Bilateral sphenoidotomies, and tumor resection was done similar to the rescue flap group. In cases where no CSF leak was observed (*n* = 23), reconstruction was done by surgicel, bone chips and free mucosal grafts. Both nasoseptal flaps were returned and stitched in their original position. When there was CSF leak (*n* = 7), the full flap was used for sellar floor reconstruction, and the smaller one was returned to its original position.

In all patients (*n* = 60), bilateral septal splints were placed at the end of the surgical procedure, and Merocel nasal packing was done in all cases.

### Post-operative care and follow up

The Merocel nasal pack was removed two days after surgery, after which patients were discharged from the hospital. Systemic antibiotics were prescribed for one week. Saline irrigation was also prescribed. The septal splints were removed one week after surgery. follow-up visits were scheduled on a weekly basis in the first one month, then on a monthly basis for 3 months.

### Outcomes

The primary outcomes were the nasal functions and morbidities after surgery. The two groups were compared regarding the nasal morbidity, posterior septal perforation, crusting, adhesions and olfaction. Assessment of these outcomes was done one month and 3 months after surgery. Secondary outcomes included the operative time, postoperative CSF leak and other postoperative complications.

Sinonasal outcome test (SNOT-22) [12] was applied to assess the overall nasal function. The validated Arabic version [13] was applied as the population of the study were Arabic speaking. Crusting was evaluated and graded using the scale that was adopted by Benzer et al. [4] where “0” denotes no crusting, “1”: mild crusting, “2”: moderate crusting, and “3”: severe crusting. Adhesions were evaluated by endoscopic examination and were quantified using the scale that was adopted by Hegazy et al. [14] on a 0–2 basis (0 = not present, 1 = present, 2 = marked).

Olfaction was evaluated using the smell diskette test that was adopted by Alotaibi et al. [15]. This test consisted of 8 smell diskettes and a questionnaire. One mark is given for each correct answer and zero mark for each wrong answer with a total mark ranging from 0 to 8.

Operative time was assessed in terms of the total operative time and the nasal stage time. The total time was the whole time from the initiation of the procedure till the insertion of the nasal packs. The nasal stage time was the time taken by the otolaryngologists in preparation and elevation of the flaps, as well as performing the reconstruction after tumor excision. This was calculated by subtracting the neurosurgeons’ tumor resection time from the total operative time. Lastly, intraoperative, and postoperative complications were recorded and analyzed.

### Statistical analysis

Data analysis was performed by SPSS software, version 26 (SPSS Inc., PASW statistics for windows version 26. Chicago: SPSS Inc.). Qualitative data were described using numbers and percentages. Quantitative data were described using median (minimum and maximum) for non-normally distributed data and mean ± Standard deviation for normally distributed data after testing normality using Kolmogrov-Smirnov test. The significance of the obtained results was judged at the (0.05) level. The Chi-Square, Fisher exact test was used to compare qualitative data between groups as appropriate. The Student T test and Mann Whitney U test were used to compare between 2 studied groups for non-normally and normally distributed data. Wilcoxon signed rank test was used to compare follow up findings between 2 studied readings of non-normally distributed data.

## Results

**Fig. 3:**
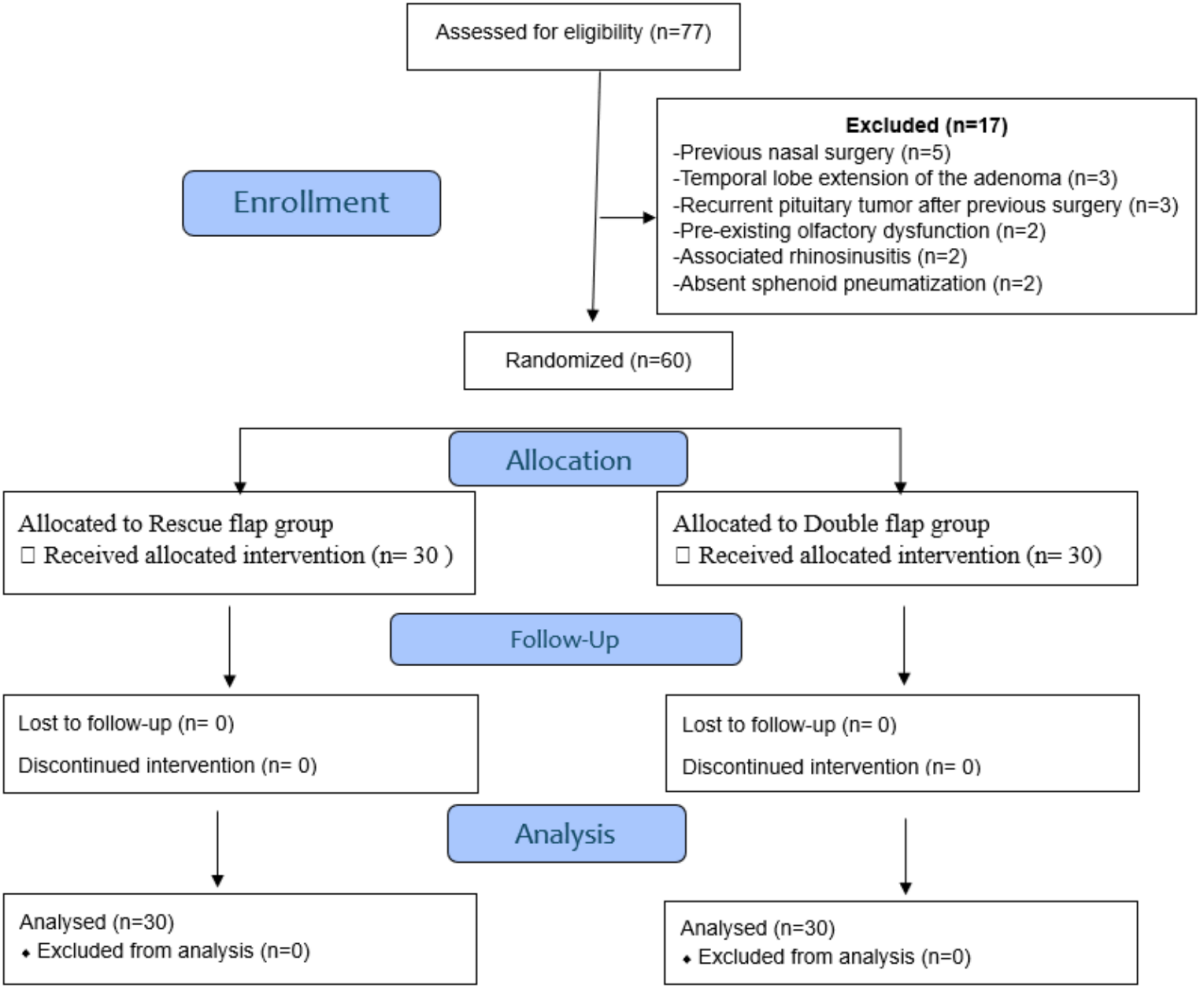
The consort flow chart of the study

Seventy-seven patients were eligible for inclusion in the current study. Seventeen patients were excluded due to previous nasal surgery (*n* = 5), temporal lobe extension of the adenoma (*n* = 3), recurrent pituitary tumor after previous surgery (*n* = 3), chronic rhinosinusitis (*n* = 2), pre-existing olfactory dysfunction (*n* = 2) and absent sphenoid pneumatization (*n* = 2). The remaining 60 patients were included and analyzed (Fig. 3).

Table (1) shows the demographic and clinical data of the included patients. both groups were matched regarding the demographic data with no statistical difference between them.

The mean volume of the pituitary adenoma was 9.11 cm^3^ in the rescue flap group and 11.06 cm^3^ in the double flap group, with no significant difference. Similarly, no significant difference between both groups was found regarding the number of functioning adenomas and the tumor extensions to the sphenoid sinus, suprasellar and cavernous sinus extensions (Table 1).

**Table 1:** Demographic and clinical data among the studied groups

		Group A (Rescue flap group) *N* = 30	Group B (Double flap group) *N* = 30	Test of significance
Age / years: Mean ± SD		47.80 ± 11.9	45.93 ± 10.62	t = 0.641 *p* = 0.524
Gender: Number (%)	**Male** **Female**	13(43.3) 17(56.7)	13(43.3) 17(56.7)	*p* = 1.0
BMI (Kg/m^2^): Mean ± SD		31.29 ± 3.05	31.72 ± 2.76	t = 0.568 *P* = 0.572
Tumor volume: cm^3^		9.11(2.85–41.89)	11.06(2.29–47.25)	Z = 0.377 *P* = 0.706
Functioning adenoma	**No** **Yes**	21(70) 9(30)	22(73.3) 8(26.7)	χ^2^=0.082 *P* = 0.774
Tumor extension	**Suprasellar Extension**	12(40)	14(46.7)	χ^2^=0.271 *P* = 0.602
	**Cavernous sinus extension**	6(20)	7(23.3)	χ^2^=0.098 *P* = 0.754
	**sphenoid sinus extension**	4(13.3)	8(26.7)	χ^2^=1.67 *P* = 0.197

t: Student t test, χ^2^: Chi-Square test, FET: Fisher exact test, Z:Mann Whitney U test, data expressed as mean ± SD or as median (min-max)

Regarding the study outcomes (Table 2), the sinonasal outcomes, assessed by the SNOT-22 questionnaire, were significantly better among the double flap group compared to the rescue flap group at 1 month and 3 months after surgery (*p* = 0.05 and 0.006 respectively). Similarly, olfaction was significantly better among the double flap group than the rescue flap group at 1 month and 3 months after surgery (*p* = 0.002 and 0.001, respectively).

Posterior septal defect/perforation was detected in 22 out of 30 patients (73.3%) in the rescue flap group, and in only 7 out of 30 patients (23.3%) in the double flap group, with a statistically significant difference (*P* < 0.001).

**Table 2:** Study outcomes

			Group A (Rescue flap group) *N* = 30	Group B (Double flap group) *N* = 30	Test of significance
SNOT	**basal**		11(3–20)	12(5–21)	Z = 0.563 *P* = 0.573
	**1 month**		26(13–57)	21(9–47)	Z = 1.96 *P* = 0.05*
	**3 months**		14(6–29)	11(5–21)	Z = 2.78 *P* = 0.006*
Olfaction score	**basal**		7.5(4–8) 7.2 ± 1.09	7(6–8) 7.33 ± 0.61	Z = 0.327 *P* = 0.744
	**1 month**		2(0–5) 2.40 ± 1.4	4(1–7) 3.70 ± 1.47	Z = 3.13 *P* = 0.002*
	**3 months**		4(1–6) 3.77 ± 1.07	6.5(4–8) 6.37 ± 1.16	Z = 5.98 *P* = 0.001*
Posterior septal defect/perforation			22(73.3)	7(23.3)	χ2 = 15.01 *P* < 0.001*
Crusting score	**1** **month**	**0** **1** **2** **3**	0 0 9(30) 21(70)	5(16.7) 9(30) 13(43.3) 3(10)	χ^2MC^=28.23 *P* < 0.001*
		**Median (Min-max)**	3(2–3)	2(0–3)	*P* < 0.001*
	**3** **months**	**0** **1** **2** **3**	0 4(13.3) 13(43.3) 13(43.3)	17(56.7) 10(33.3) 2(6.7) 1(3.3)	χ^2MC^=37.92 *P* < 0.001*
		**Median (Min-max)**	2(1–3)	0(0–3)	*P* < 0.001*
Adhesions score	**0** **1** **2**		10(33.3) 15(50) 5(16.7)	10(33.3) 19(63.3) 1(3.3)	χ^2^ =3.14 *P* = 0.208
Operative time	**Total Operative Time**		145.67 ± 31.12	155 ± 23.92	t = 1.30 *p* = 0.198
	**Nasal Stage Time**		83.17 ± 15.39	97.33 ± 10.65	t = 4.15 *p* = 0.001*
Intraoperative CSF Leak			6(20)	7(23.3)	χ^2^ =0.098 *P* = 0.754
Sellar reconstruction	No Yes		24(80) 6(20)	23(76.7) 7(23.3)	χ2 = 0.098 *P* = 0.754
Rate of intraoperative CSF Leak	**Low** **High**		3(50) 3(50)	3(42.9) 4(57.1)	χ^2FET^=0.07 *P* = 1.0
Postoperative CSF leak			1(16.7)	1(14.3)	FET = 0.014 *P* = 1.0
Postoperative epistaxis			3(10)	2(6.7)	χ^2FET^ =0.218 *P* = 1.0

χ^2^:Chi-Square test, t: Student t test, Z:Mann Whitney U test, FET: Fisher exact test, *statistically significant (*p* < 0.05)

Crusting was significantly more severe among the rescue flap group than the double flap group at 1 month and 3 months after surgery (*p* < 0.001). significant adhesions occurred among the rescue flap group patients more frequently than the double flap group, however the difference was not satisfactory significant (*p* = 0.2).

As regards the nasal stage operative time, it was significantly longer in the double flap group than the rescue flap group (*p* = 0.001), as the mean time was 97.33 and 83.17 min, respectively.

Intraoperative CSF leak that required performing sellar floor reconstruction occurred in 6–30 patients (20%) in the rescue flap group and in 7/30 patients (23.3%) in the double flap group, with no statistically significant difference (*p* = 0.754).

No intraoperative complications were reported in the current work. Postoperative complications were reported in the form of CSF leak in 2 patients (one in the rescue flap group and one in the double flap group). This was successfully managed with conservative and medical measures (bed rest with head elevation and acetazolamide treatment). None of the patients required surgical intervention. Additionally, minor epistaxis occurred in 5 patients (3 patients in the rescue flap group and 2 in the double flap group). Epistaxis was controlled in all patients with conservative measures (compression, local moisturizing treatment, saline sprays and hemostatic medications) without the need for nasal packing or surgical control.

Follow up duration ranged from 6 to 24 months (mean 17.5 months). No other treatment related side effects were reported during the follow up period. The patients were referred to the endocrinologists for hormonal and replacement therapies as needed.

## Discussion

Endoscopic endonasal pituitary surgery may negatively impact the sinonasal functions [16]. Gstrein et al. [1] performed a recent large meta-analysis in 2023 that included 1533 patients who underwent endoscopic trans-sphenoid pituitary surgery and reported a median postoperative epistaxis rate of 1.4%, a postoperative acute rhinosinusitis rate of 2.3%, a postoperative synechiae rate of 7.5%, and a postoperative septal perforation rate of 2.2%. Similarly, in their review article, Awad et al. [17], found that nasal crusting occurred in 50.8% of patients, followed by nasal discharge (40.4%), nasal airflow blockage (40.1%), and disturbances in olfaction (26.7%). In the current study, epistaxis occurred in 8.3%, marked adhesions in 10%, and severe crusting in 23.3%.

The current study demonstrated that the double nasoseptal flap group showed better postoperative nasal functions in terms of better SNOT-22 scores, better olfaction scores, less crusting, less adhesions and less incidence of septal perforation. These findings can be explained by better mucosal preservation, compared to the rescue flap group, where the mucosal of the posterior septum, contralateral to the flap is resected. In a similar study, Benzer et al. [4] assessed the sinonasal outcomes of the double nasoseptal flap compared to the rescue flap. Their findings indicated that patients in the double flap group experienced superior results regarding nasal breathing, crusting, and the formation of synechiae.

Interestingly, Benzer et al. [4] reported that intact septum was seen in all patients (30) in the double nasoseptal group, and posterior septal perforation was seen in all patients (30) in the rescue flap group. Furthermore, they demonstrated that posterior septal perforation was associated with worse nasal symptoms evaluated through a visual analogue scale. The authors attributed these adverse outcomes to the mucosal loss in the posterior parts of the nasal septum.

The authors of the current work adopted the double nasoseptal flap approach in order to perform the posterior septectomy by resection of the septal bone (ethmoid plate and vomer) with bilateral mucosal preservation. Some authors described a reverse rotation flap technique [18] which involves performing a posterior septectomy while preserving the contralateral septal mucosa. The contralateral mucosa is then incised to create an anteriorly based mucosal flap that is then draped over the ipsilateral donor site. In the current study the preserved contralateral flap was returned to its original site to maintain the integrity of the nasal septum and to minimize risk of septal perforation.

Excess mucosal resection is a key factor in nasal morbidity, especially olfactory dysfunction. The olfactory neuroepithelium is known to line the cribriform plate, superior turbinate, superior septum, and some areas of the middle turbinate [19]. Multiple studies have found that endoscopic pituitary surgery may result in significant olfactory dysfunction due to mucosal resection [3, 20].

Some researchers have shown significant loss of olfactory function associated with the use of the nasoseptal flap for reconstruction [20, 21], while others reported that the postoperative smell function was not impaired after reconstruction with nasoseptal flap [1]. In our study, satisfactory olfactory function scores (almost returned to the basal scores) were obtained at 3 months after surgery in the double nasoseptal flap group. One of the possible contributing factors to the preserved olfactory function is preservation of the olfactory mucosa in the nasal septum by placing the superior incision at least 1 cm. below the skull base [22].

Olfactory strip preservation is another technique adopted by some authors [23–25] to improve postoperative olfaction. In this technique, the superior incision starts at the level of the sphenoid natural ostium and continues anteriorly onto the nasal septum 1.5–2 cm below any identifiable olfactory epithelium. The olfactory strip is visually identified based on the fact that the olfactory epithelium typically has a characteristic yellowish hue, and its vasculature follows an axial pattern. In the current study, however, the superior incision was made 1.5–2 cm below the skull base without relying on the visual identification of the olfactory mucosa which is not reliable in all patients, and good olfactory outcomes were achieved, close to the baseline preoperative scores in the double flap group.

Another critical factor is middle turbinate preservation. Previous studies showed the impact of middle turbinate removal on the incidence sinonasal morbidity [3, 26]. The middle turbinate is crucial for humidifying, filtering, and regulating the temperature of air before it enters the lower airway tract. Historically, many surgeons sacrificed the middle turbinate to improve exposure and surgical access during endonasal skull base surgery [27, 28]. Nyquist et al. [26] concluded that that preserving the middle turbinate is associated with better sinonasal function while still providing good surgical access. In the current study the middle turbinate was preserved in all patients (*n* = 60) with adequate surgical exposure.

In the current study, the incidence of posterior septal perforation was significantly higher in the rescue flap group than the double nasoseptal flap group. Septal perforation is uncommon with the nasoseptal flap, however it may be related to cartilage necrosis or injury to the contralateral vascular supply to the septum during the procedure [8].

Postoperative epistaxis was reported in 8.3% in this work. Similarly, Awad et al. [17] demonstrated that epistaxis is uncommon after endoscopic skull base surgery. Thompson et al. [29] reported an incidence of 3% in 330 patients within 30 days of endoscopic skull base surgery or various pathologies. Other rare complications were reported in the literature such as delayed epithelialization of the denuded areas with granuloma formation, sinonasal mucoceles, external nose deformity such as saddle nose, nasoseptal flap necrosis and meningitis [22, 30, 31]. None of these complications were reported in this study.

A possible disadvantage of the double nasoseptal flap technique is the longer nasal stage operative time in comparison to the rescue flap technique. Longer time is needed for elevation of bilateral flaps and performing reconstruction after tumor resection or returning the flaps to their original site if there is no CSF leak. Nevertheless, the authors of this work believe that its major advantage regarding better sinonasal functional outcomes outweighs this disadvantage. Another potential drawback of raising two nasoseptal flaps upfront would be decreased ability to customize the nasoseptal flap size depending on the ultimate defect. This consideration becomes particularly important during the excision of large pituitary adenomas with significant suprasellar extensions, as well as in the management of other extensive skull base tumors.

## Conclusion

Endoscopic pituitary surgery can adversely affect the sinonasal functions. Double nasoseptal flap technique allows posterior septectomy with bilateral septal mucosa preservation. Although it requires longer operative time than the rescue flap technique, better functional sinonasal outcomes and olfaction scores are achieved.
